# Finite-Size Effects in Simulations of Peptide/Lipid Assembly

**DOI:** 10.1007/s00232-022-00255-9

**Published:** 2022-07-19

**Authors:** Zack Jarin, Olivia Agolini, Richard W. Pastor

**Affiliations:** Laboratory of Computational Biology, National Heart, Lung, and Blood Institute, National Institutes of Health, Bethesda, MD USA

**Keywords:** Self-assembly, Molecular dynamics, Coarse-grained, Amphipathic peptides, Finite-size effects

## Abstract

**Abstract:**

Molecular dynamics simulations are an attractive tool for understanding lipid/peptide self-assembly but can be plagued by inaccuracies when the system sizes are too small. The general guidance from self-assembly simulations of homogeneous micelles is that the total number of surfactants should be three to five times greater than the equilibrium aggregate number of surfactants per micelle. Herein, the heuristic is tested on the more complicated self-assembly of lipids and amphipathic peptides using the Cooke and Martini 3 coarse-grained models. Cooke model simulations with 50 to 1000 lipids and no peptide are dominated by finite-size effects, with usually one aggregate (micelle or nanodisc) containing most of the lipids forming at each system size. Approximately 200 systems of different peptide/lipid (P/L) ratios and sizes of up to 1000 lipids yield a “finite-size phase diagram” for peptide driven self-assembly, including a coexistence region of micelles and discs. Insights from the Cooke model are applied to the assembly of dimyristoylphosphatidylcholine and the ELK-neutral peptide using the Martini 3 model. Systems of 150, 450, and 900 lipids with P/L = 1/6.25 form mixtures of lipid-rich discs that agree in size with experiment and peptide-rich micelles. Only the 150-lipid system shows finite-size effects, which arise from the long-tailed distribution of aggregate sizes. The general rule of three to five times the equilibrium aggregate size remains a practical heuristic for the Cooke and Martini 3 systems investigated here.

**Graphical Abstract:**

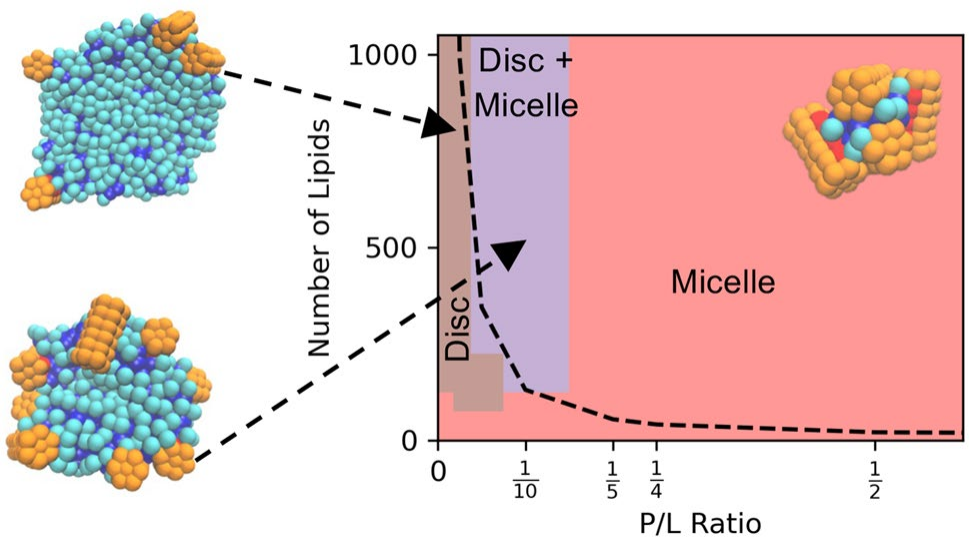

**Supplementary Information:**

The online version contains supplementary material available at 10.1007/s00232-022-00255-9.

## Introduction

In principle, the optimal micelle size balances the attraction of hydrophobic tails and the repulsion of headgroups, which are both a function of the number of surfactants in the micelle. (Tanford, 1974) However, molecular simulations of self-assembly with insufficiently large system sizes produce unreliable results which are often revealed by large discrepancies in aggregation numbers and size distributions among simulations of different sizes. In their detailed treatment of finite-size effects in micelle self-assembly by surfactants Straub and coworkers (Harris et al., 2021) provide a useful piece of advice for simulations with only one (non-solvent) component: the preferred aggregation number can be reliably calculated when the system size is at least three times larger than the equilibrium aggregation number.

Lipid/peptide and long-tail/short-tail lipid mixtures which form discoidal aggregates and mixed micelles at low lipid concentrations are both systems requiring formulation of a two-component heuristic. This paper considers system size requirements and finite-size effects on the aggregation of dimyristoylphosphatidylcholine (DMPC) and the amphipathic peptide ELK-neutral (ELK-neu). ELK peptides are composed of only Glu, Leu, and Lys, and have been shown to solubilize lipids, stabilize discoidal structures at a ratio near 5 lipids to 1 peptide, and to efflux cholesterol. (Islam et al., 2018) Thus, ELK peptides (and synthetic amphipathic peptides in general) are a possible component of a promising drug delivery vehicle and a potential cardiovascular disease therapeutic. (Ditiatkovski et al., 2013; Islam et al., 2018; Ossoli et al., 2022) ELK-neu (EKLKELLEKLLEKLKELL) is a particularly effective member of the set. The preceding applications depend on the aggregation of peptides and lipids and associated molecular simulations are likely susceptible to finite-size effects.

Because exploring the aggregation of lipid/peptide nanodiscs is not feasible with all-atom simulations, two coarse-grained (CG) models are used to study the self-assembly of ELK-neu and DMPC. First, the assembly properties are modeled at a range of peptide to lipid (P/L) ratios using the low-resolution Cooke-Kremer-Deserno model and termed the Cooke model here. (Cooke & Deserno, 2005; Cooke et al., 2005) Given that an ELK-neu nanodisc contains about 1 peptide to 5 lipids (P/L = 1/5), systems with peptide to lipid ratios between 1/2 and 1/20 are investigated quantitatively, while the peptide-free system and lower peptide to lipid ratios of 1/40 and 1/100 are considered for qualitative analysis. For the parameters simulated, the lipid-only systems form very large discs, and determination of average size is not in the scope of this paper. Nevertheless, it is shown definitively that the peptide-free discs are substantially larger than those formed when peptides are present. Indeed, at high P/L ratios, discs are not stable and micellar assemblies are found instead. Next, the recent Martini 3 model (Souza et al., 2021) is employed to contextualize insights from simulations using the Cooke model to P/L ratios of interest. The recent update to the Martini model is necessary for this study given over-aggregation of proteins and peptides in the previous version. (Jarin et al., 2021; Javanainen et al., 2017; Periole et al., 2018; Stark, Andrews & Elcock, 2013) The Martini 3 model has improved upon the previous iteration with updated mapping heuristics and an expanded set of bead types that includes more bead sizes and interaction strengths. While the accuracy of the Martini model will be discussed, the primary goal of this study is to use it to examine finite-size effects in this specific example of lipid/peptide self-assembly.

Approximately 200 different simulation sizes/compositions and over 1000 individual trajectories of length 10–65 μs were carried out for this study. The first two subsections of the Results describe Cooke model simulations of pure lipid and lipid/peptide assembly, respectively. They yield clear examples of finite-size effects, provide a test of previous heuristics to avoid finite-size effects, and allow the construction of a “finite-size driven phase diagram”. The third subsection presents Martini 3 model simulations of self-assembly of DMPC and ELK-neu at three different system sizes. These are analyzed with respect to the Cooke model results and compared with all-atom simulations and experiments.

## Methods

The Cooke model is a low-resolution CG lipid model with only a few bead types and no explicit water. (Cooke & Deserno, 2005; Cooke et al., 2005; Deserno, 2009) It has been successfully applied to investigate self-assembly processes including examples of bicelle formation (Vacha & Frenkel, 2014), lipid/peptide self-assembly (Midtgaard et al., 2014), and pore formation in membranes. (Illya & Deserno, 2008) In these examples, the short-/long-tailed lipids assembled into micelles, worm-like micelles, and bicelles, the peptide/lipids formed mixtures of peptide-rich fibers, mixed micelles, and discs, and the trans-bilayer peptides assembled into pores. The first two are two-component assembly processes and the last is an example of single-component self-assembly in the bilayer environment. If solvent were explicitly represented (e.g., Martini 3 model), it would not be considered a component of the assembled structure.

**Fig. 1:**
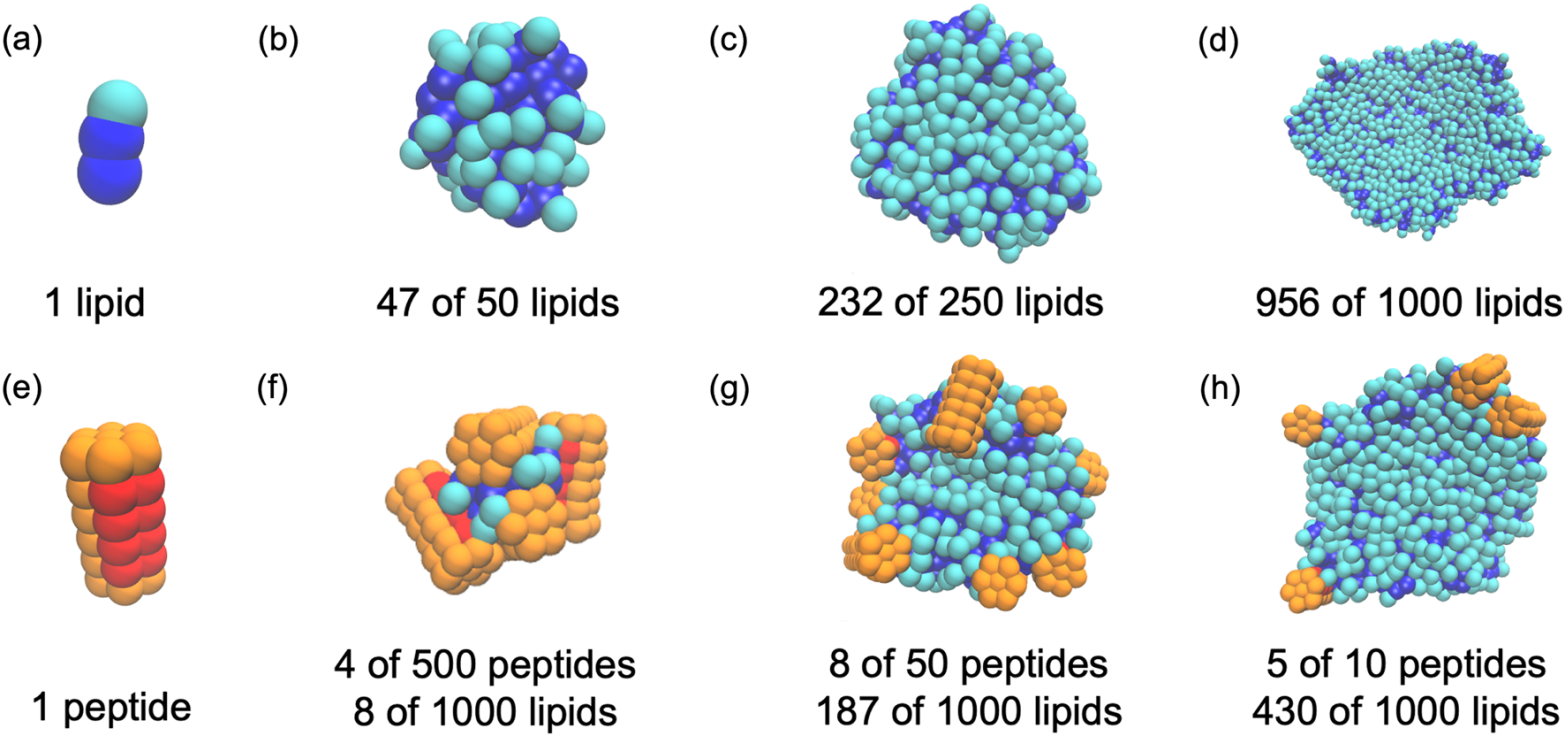
Representative snapshots from Cooke model simulations. **a** individual lipid, **b** lipid micelle composed of 47 lipids in a 50-lipid system, **c** lipid disc of 232 lipids in a 250-lipid system, **d** lipid disc of 956 lipids in a 1000-lipid system **e** an individual peptide, **f** mixed micelle composed of 4 peptides and 8 lipids in a 500 peptide/1000-lipid system, **g** disc composed of 8 peptides and 187 lipids in a 50-peptide/1000-lipid system, and **h** disc composed of 5 peptides and 430 lipids in a 10-peptide/1000-lipid system

This study uses a 3-bead lipid model with two hydrophobic beads (dark blue in Fig. 1a) and a single hydrophilic bead (light blue), and a 42-bead peptide model with eight hydrophobic beads (red in Fig. 1e) and 34 hydrophilic beads (orange) arranged in a stack of 6 filled hexagons. Previous studies (Schindler et al., 2016) provided examples of lipid representations with similar interaction parameters. In particular, a 3-bead lipid model with the parameters used here was shown to form a fluid bilayer with experimentally and biologically relevant bending stiffness and compression modulus but does not form a closed structure (i.e., a vesicle). The compatible ELK-neu representation is chosen to capture the hydrophobic surface of a folded ELK-neu in the membrane environment. Comparing the new ELK-neu model to the previous amphipathic Cooke peptides, the ELK-neu model is shorter to be compatible with a 3-bead lipid rather than the 4-bead lipid used in previous studies. Hence, it has fewer hydrophobic beads than the pore-forming peptide. (Illya & Deserno, 2008) The ELK-neu model has additional hydrophilic ends compared to the double-belt forming peptide. (Midtgaard et al., 2014) These changes result in a peptide and lipid model which assembles into mixed micelle and disc-like aggregates.

The Cooke force field is composed of two nonbonded terms accounting for repulsion and attraction and two bonded terms governing the bonds and angles. The repulsive potential, given by the Weeks-Chandler-Andersen potential, controls the size of hydrophobic (T) and hydrophilic (H) beads:

1 $$V_{{rep}} \left( {r;b} \right) = \left\{ {\begin{array}{*{20}c} {4\varepsilon \left[ {\left( {b/r} \right)^{{12}} - \left( {b/r} \right)^{6} + 1/4} \right],} & {r \le r_{c} } \\ {0,} & {r{\text{ > }}r_{c} } \\ \end{array} } \right.$$

where ${r}_{c}={2}^{1/6}b$_,_ ${b}_{HH}={b}_{HT}=0.95\sigma$, and ${b}_{TT}=\sigma$. (Weeks et al., 1971) $\sigma$ and $\epsilon$ are the characteristic length and energy scales of the Cooke model and are chosen to be 0.6 nm and 1 kcal/mol, respectively. The difference in hydrophilic and hydrophobic bead sizes is to maintain a cylindrical lipid shape. The attractive nonbonded term (Eq. (2)) is a piecewise function, which over a distance of ${w}_{c},$ smoothly transitions from $-\epsilon$ to 0 using a cosine squared function.

2 $$V_{att} \left( r \right) = \left\{ {\begin{array}{*{20}c} { - \varepsilon ,} \\ {\varepsilon \cos^{2} \left[ {\pi \left( {r - r_{c} } \right)/\left( {2w_{c} } \right)} \right]} \\ {0,} \\ \end{array} } \right.,\begin{array}{*{20}c} {r < r_{c} } \\ {r_{c} \le r \le r_{c} + w_{c} } \\ {r > r_{c} + w_{c} } \\ \end{array}$$

where ${w}_{c}$=$\sigma$ for lipid-lipid and lipid-peptide hydrophobic-hydrophobic interactions and 0 for all other cases (i.e., peptide-peptide hydrophobic-hydrophobic or hydrophilic-hydrophilic). The peptide-peptide attraction is set to 0 because aqueous ELK-neu is less structured than in the membrane environment and ELK-neu alone does not aggregate. (Islam et al., 2018).

The bonded terms of the Cooke lipid model are shown in Eqs. (3) and (4). The bond potential of the lipids is governed by two finite extensible nonlinear elastic (FENE) bonds,

3 $$V_{bond} \left( r \right) = - \left( {1/2} \right)k_{bond} r_{\infty }^{2} {\text{log}}\left[ {1 - \left( {r/r_{\infty } } \right)} \right]^{2}$$

where bond stiffness ${k}_{bond}=30\epsilon /{\sigma }^{2}$ and divergence length ${r}_{\infty }=1.5\sigma$. The linearity of the lipid is maintained by a harmonic bond between head and second tail bead.

4 $$V_{bend} \left( r \right) = \left( {1/2} \right)k_{bend} \left( {r - 4\sigma } \right)^{2}$$

where bending stiffness ${k}_{bend}=10\epsilon /{\sigma }^{2}$ and equilibrium length of $4\sigma$. While the lipid structure is rather flexible, the structure of ELK-neu is maintained by a fully connected elastic network between beads of adjacent layers with bond stiffness $k=30\epsilon /{\sigma }^{2}$. For example, a bead in the hydrophilic cap at the end of the peptide will have 13 total bonds: six to the other beads of that layer and seven to the beads in the next layer. The high connectivity and strong bond constant keep the shape and structure of ELK-neu nearly rigid.

All Cooke model simulations were run in OpenMM (Eastman et al., 2017) in the NVT ensemble at 310 K using a time step of 10 fs and a Langevin thermostat dampening of 1 ps^−1^. Random initial configurations in cubic simulation cells were generated using PACKMOL. (Martinez et al., 2009) Overlapping beads were equilibrated by running energy minimization, followed by short Langevin dynamics simulations with increasing time step starting at 0.01 fs. Production runs were run using a 10 fs time step and Langevin dampening coefficient of 1 ps^−1^. Eight P/L ratios were simulated: 0, 1/100, 1/40, 1/20, 1/10, 1/5, 1/4, and 1/2. Based on the phase and finite-size behavior, the eight P/L ratios can be separated into low (P/L = 0 and 1/100), intermediate (1/40, 1/20, 1/10), and high (1/5, 1/4, 1/2). All simulations were run between 17.5 and 37.5 μs. See SI Tables 1–3 for a full list of systems and corresponding contents, sizes, and simulation lengths.

The Martini 3 model was used for more realistic simulations of ELK-neu and DMPC. The Martini family of models uses an approximate four-to-one heavy atoms to CG bead; water is explicit and also follows the four-to-one mapping. While the Martini 3 model has not been applied to lipid/peptide self-assembly studies to date, the latest Martini model builds upon the Martini 2’s successes by expanding the parameter set and improving the peptide-peptide interactions. (Souza et al., 2021) All Martini simulations were run in GROMACS with a harmonic restraint enforcing helical secondary structure. (Abraham et al., 2015; Berendsen et al., 1995) Random initial configurations in cubic simulation cells were generated using PACKMOL. (Martinez et al., 2009) System equilibration was performed as follows: steepest descent energy minimization which ends after 5000 steps or when the maximum force on a particle is less than 10 kJ mol^−1^ nm^−1^; 500 ps of NVT with time step of 1 fs and temperature control by velocity rescaling with a coupling of 1 ps^−1^; 2 ns of NPT with time step of 1 fs, temperature coupling of 1 ps^−1^ and Berendsen barostat with coupling of 5 ps^−1^; 5 ns of NPT with a time step of 1 fs, temperature coupling of 1 ps^−1^, and Parrinello-Rahman barostat coupling of 5 ps^−1^. (Berendsen et al., 1984; Bussi et al., 2007; Parrinello & Rahman, 1981) Since 30 individual Martini 3 simulations were run, a conservative equilibration scheme with an atypically low time step was used to avoid system crashes arising from poor packing. Production simulations were carried out with a 10 fs time step, temperature coupling 1 ps^−1^, and Parrinello-Rahman pressure coupling 12 ps^−1^. Trajectories were generated for systems of 150, 450, and 900 lipids for 30, 40, and 65 μs, respectively, with 10 replicates for each. Production simulations of the 900-lipid system generated 60 ns/day on a single GPU, which is 40–50 times slower than the analogous Cooke system. Concentrations were ~ 7 mM for peptides, ~ 42 mM for lipids, and ~ 150 mM for NaCl. The lipid to peptide ratio of 6.25 is based on experimental measurements of ELK-neu in a 1-palmitoyl-2-oleoyl-glycero-3-phosphocholine (POPC)/cholesterol mixture (Islam et al., 2018), and is assumed to be the same for the peptide in DMPC. A POPC/cholesterol mixture could lead to larger lipid/peptide aggregates given POPC has longer acyl chains than DMPC and cholesterol is not included in the present simulations. In the absence of a precise experimental composition, the size of self-assembled ELK-neu/DMPC is compared to size measurements from gel electrophoresis. (Islam et al., 2018).

Simulation analysis and figures used the python packages: NumPy (Harris et al., 2020), Matplotlib (Hunter, 2007), MDTraj (McGibbon et al., 2015), and Freud (Ramasubramani et al., 2020). Clustering analysis used a cutoff of 0.9 nm for the Cooke model and 0.7 nm for the Martini 3 model and was only performed on the hydrophobic beads to reduce the number of artificial aggregation events as aggregates move within a cutoff of each other. The number of aggregates, radius of gyration of aggregates using all beads, and aggregate composition are reported for aggregates containing at least 5 total lipids or peptides. A radius of gyration cutoff of 2.5 and 2 nm was used to differentiate disc-like and micelle-like aggregates for the Cooke and Martini 3 models, respectively. While the radius of gyration is a suitable delimiter between micelle and disc aggregates, it is a poor approximation of the size. Instead, the size of the aggregate is calculated as the average of the range in the first two principal components, which is a good approximation of the diameter of a spherical micelle or diameter of a cylindrical disc. The mean square deviation of the number of aggregates between replicas as a function of time was used as a measure of ergodicity and is reported for the 1000-lipid Cooke systems (Fig. S1) and the Martini 3 (Fig. S3) systems in the Supplemental Information. (Thirumalai & Mountain, 1990; Thirumalai et al., 1989).

## Results

### Cooke Model: Lipids Only

The Cooke model forms a variety of morphologies dependent on system size. The radius of gyration of each aggregate is used to distinguish aggregates as micelle-like and disc-like, and, subsequently, to gain insight to the phase as a function of system size. Panels b-d of Fig. 1 show the peptide-free lipid aggregates. In a 50-lipid system (Fig. 1b), a single micelle formed, which was composed of nearly all the lipids in the system. Here, the system size obviously limits the size of the aggregate and the resulting phase. This is a clear and simple demonstration of a simulation size affecting the phase behavior of the simulation. With increasing system size, aggregates increase in size and transition to disc-like structures as seen in Fig. 1c and d in agreement with previous self-assembly simulations of the Cooke lipids. (Schindler et al., 2016) Indeed, all the pure lipid systems modeled here have finite-size effects, and simulations with more lipids simply generate larger aggregates. The system size required to generate multiple nanodiscs in the same simulation (and thereby estimate the average size) was not determined.

**Fig. 2:**
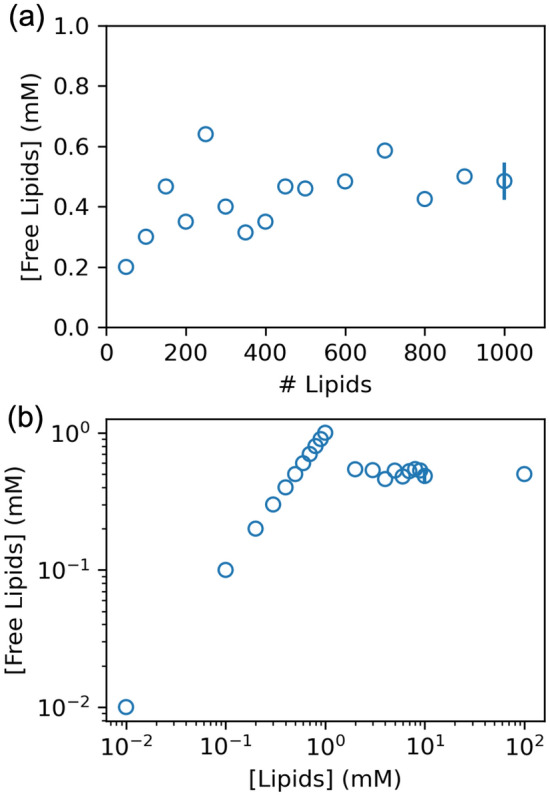
Estimation of the critical micelle concentration from the concentration of free lipids as a function of number of lipids in the system at total lipid concentration of 10 mM (**a**), and concentration of all lipids (**b**)

While aggregate size of pure lipids is affected by finite-size effects in all systems investigated, it is still possible to determine the critical micelle concentration (CMC) from the present simulations. Figure 2a plots the concentration of free lipids vs. the number of lipids in the system, and thereby provides an estimate of the CMC when the lipid number is sufficiently large. In all simulations, the total lipid concentration is 10 mM and free lipids exchange with the one or two aggregates in the system (see Table S1 for pure lipid simulations and Figure S2a for the number of aggregates and average lipids per aggregate). The concentration of free lipids increases from the artificially low estimate in the 50-lipid system and then plateaus; the CMC estimated from the 1000-lipid system is 0.48 ± 0.06 mM.

Figure 2b shows an estimation of the CMC from a different set of simulations exploring the thermodynamic property while controlling for finite-size effects. Here, 1000-lipid systems range from 10^–2^ to 10^2^ mM (Table S2); the 10 mM simulations are the same 1000-lipid simulations in Fig. 2a. The lowest concentrations are far below the CMC, no aggregates form, and the free lipid concentration is equal to the total lipid concentration. Indeed, up to 1 mM, only short-lived aggregates of less than 10 lipids form. Micelles consistently form at 2 mM and above. Hence, the CMC can be estimated from concentrations between 2 and 100 mM (the plateau in Fig. 2b) to be 0.50 mM, which is consistent with the estimate from Fig. 2a. Based on this estimate of CMC, the systems between 0.5 and 1 mM are slightly supersaturated. From previous simulations of supersaturated vapors of Lennard–Jones particles and the application of classical nucleation theory, the nucleation rate of supersaturated vapors is slow when the density of the supersaturated vapor is near the density of the liquid and simulations of direct nucleation are plagued by long-time requirements. (Laasonen et al., 2000; Yasuoka & Matsumoto, 1998) The free Cooke lipids are expected to have similar timescale requirements because of the implicit representation of the solvent and similarity of the pairwise potential. Thus, the supersaturated concentrations between 0.5 and 1 mM likely require exceedingly long simulations to produce a stable aggregate. Nevertheless, the finite-size effects in the estimation of CMC are far less pronounced than aggregation into micelles and discs presented above and for the other finite-size effects discussed below, though care must be taken to avoid conditions that lead to supersaturation.

### Cooke Model: Lipids and Peptides

The Cooke model as parametrized here forms mixed micelles and discs depending on the size and P/L ratio of the system. Panels f–h of Fig. 1 show representative snapshots of mixed micelles and discs from 1000-lipid systems. A micelle is the highest P/L ratio aggregate that can form because the aggregate cannot accommodate any more peptides and any fewer lipids destabilize the aggregate because the peptides do not have direct attraction (see Methods). In contrast, the discs are lipid-rich aggregates where the peptide sorts to the edge of the aggregate, which resembles the all-atom behavior of ELK peptides. (Islam et al., 2018; Pourmousa & Pastor, 2018) What follows is the assessment and characterization of finite-size effects in two-component assembly using the Cooke model.

**Fig. 3:**
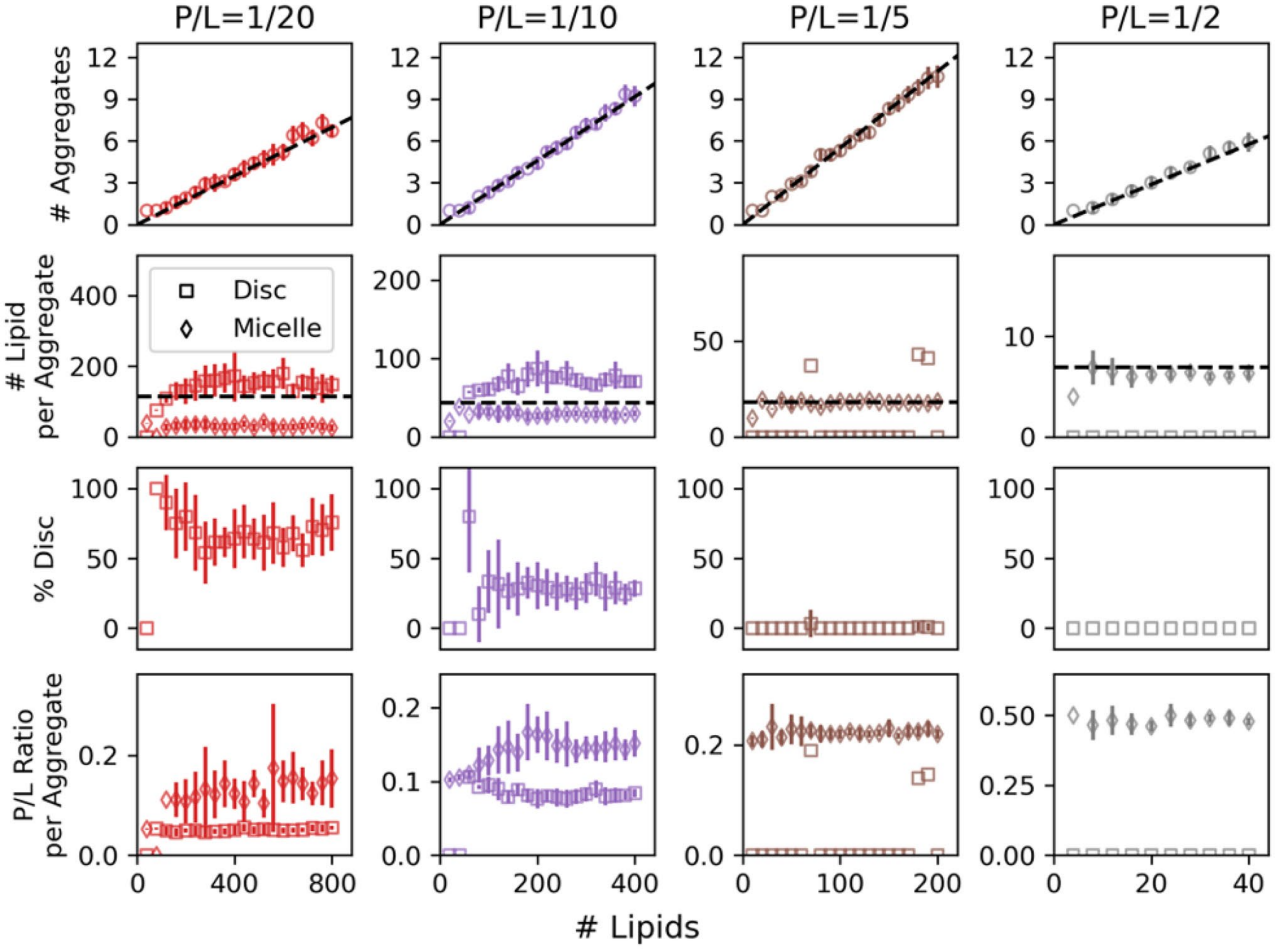
Number of aggregates, number of lipids per disc (square) or micelle (diamond), percent of aggregates that are discs, and peptide-lipid ratio per aggregate as a function of number of lipids (and peptides) for four bulk peptide/lipid ratios: 1/20 (red), 1/10 (purple), 1/5 (brown), and 1/2 (grey). Error bars are the standard deviation from 10 replicas. No error bars are shown when either all replicates yield the same value, or when there was only one aggregate of that kind

The finite-size effects of four P/L ratios are assessed first. At constant concentration (10 mM lipids), ten replicas for a range of system sizes are simulated to determine the number of aggregates, the number of lipids per aggregate, the percent of the aggregates that are disc-like, and the P/L ratio of each aggregate, shown in Fig. 3. P/L = 1/2 is presented first because it quickly overcomes finite-size effects. As shown in Fig. 3 (rightmost column) system sizes are sampled in intervals of 4 lipids from 4 to 40 (Table S3). The smallest size of four lipids and two peptides is specifically built to have finite-size effects. All systems eight lipids and larger form mixed micelles and do not exhibit finite-size effects: the number of aggregates scales linearly with the total number of lipids, while the number of lipids per aggregate, and the P/L ratio per aggregate are independent of system size. No disc-like aggregates (open squares in Fig. 3) are formed at this P/L ratio.

The systems with P/L ratio of 1/5 behave similarly to those with P/L = 1/2. However, in this case, which is sampled every 10 lipids up to 200 lipids (Table S3), the equilibrium micellar aggregate is larger, resulting in finite-size effects for system sizes less than 20 lipids. With increasing system size, the number of aggregates in the system increases in discrete steps with corresponding oscillatory behavior in the number of lipids per aggregate. In the total of 200 simulations at P/L = 1/5, micelles are the dominant aggregate and only three discs formed. There is clearly a propensity for micelle formation, but these rare discs demonstrate the need for replicas when modeling self-assembly processes.

P/L = 1/10 and 1/20 are the most complicated. For P/L = 1/20, a single micelle forms in the smallest system size (40 lipids), and a single disc forms in the second smallest (80 lipids). Hence, the percent disc jumps from 0 to 100, which is a clear finite-size effect. After overshooting the preferred percent of disc-like aggregates, P/L = 1/10 and 1/20 decay to and oscillate around about 30% and 60% disc-like aggregates, respectively, with increasing system size. This occurs in systems generating at least three aggregates. The discs that form are lipid-rich as compared to the peptide-rich micelles and the P/L per aggregate plateaus with increasing system size.

**Fig. 4:**
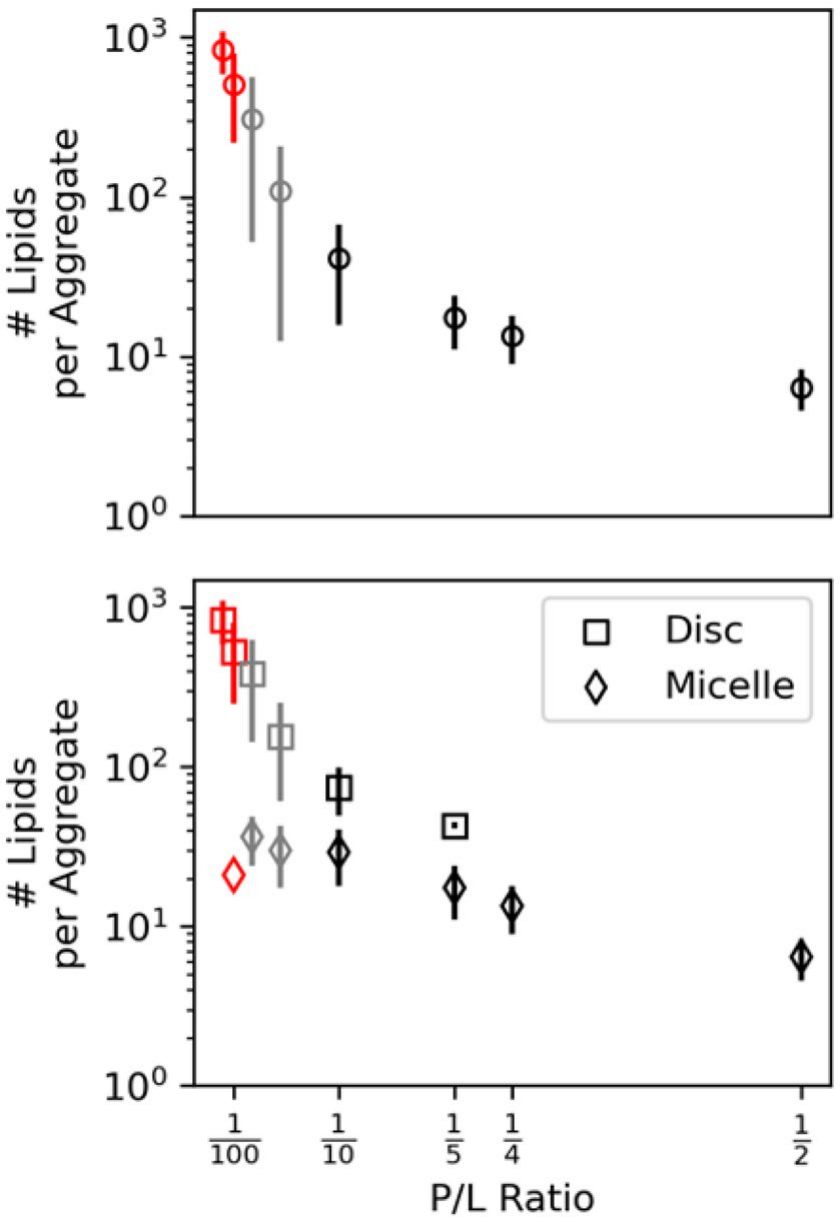
Number of lipids per aggregate using all aggregates (top) and separating discs and micelles (bottom) for eight P/L ratios from 15 replicas of 1000-lipid systems. The systems with definite, minimal, and no finite-size effects are shown in red, grey, and black

Overall, the peptide solubilizes lipids and the average aggregate size decreased with increasing P/L as seen in Fig. 4 top. The average size is calculated from 15 replicas of 1000-lipid systems for the eight P/L ratios after the number of aggregates in the simulations has converged. For the full list of simulations and the time series behavior, see Table S1 and Figure S1. At low P/L, large discs were dominant and less than three formed in each simulation; these are shown in red in Fig. 4. The systems with intermediate P/L that formed a mixture of micelles and discs are of key interest because these systems have slight finite-size effects and are shown in grey in Fig. 4. The low and intermediate P/L cases are in clear contrast to the high P/L ratio systems where many micelles formed in a single system and there are no finite-size effects. Increasing P/L clearly leads to smaller aggregates and the emergence of mixed micelles (Fig. 4 bottom).

**Fig. 5:**
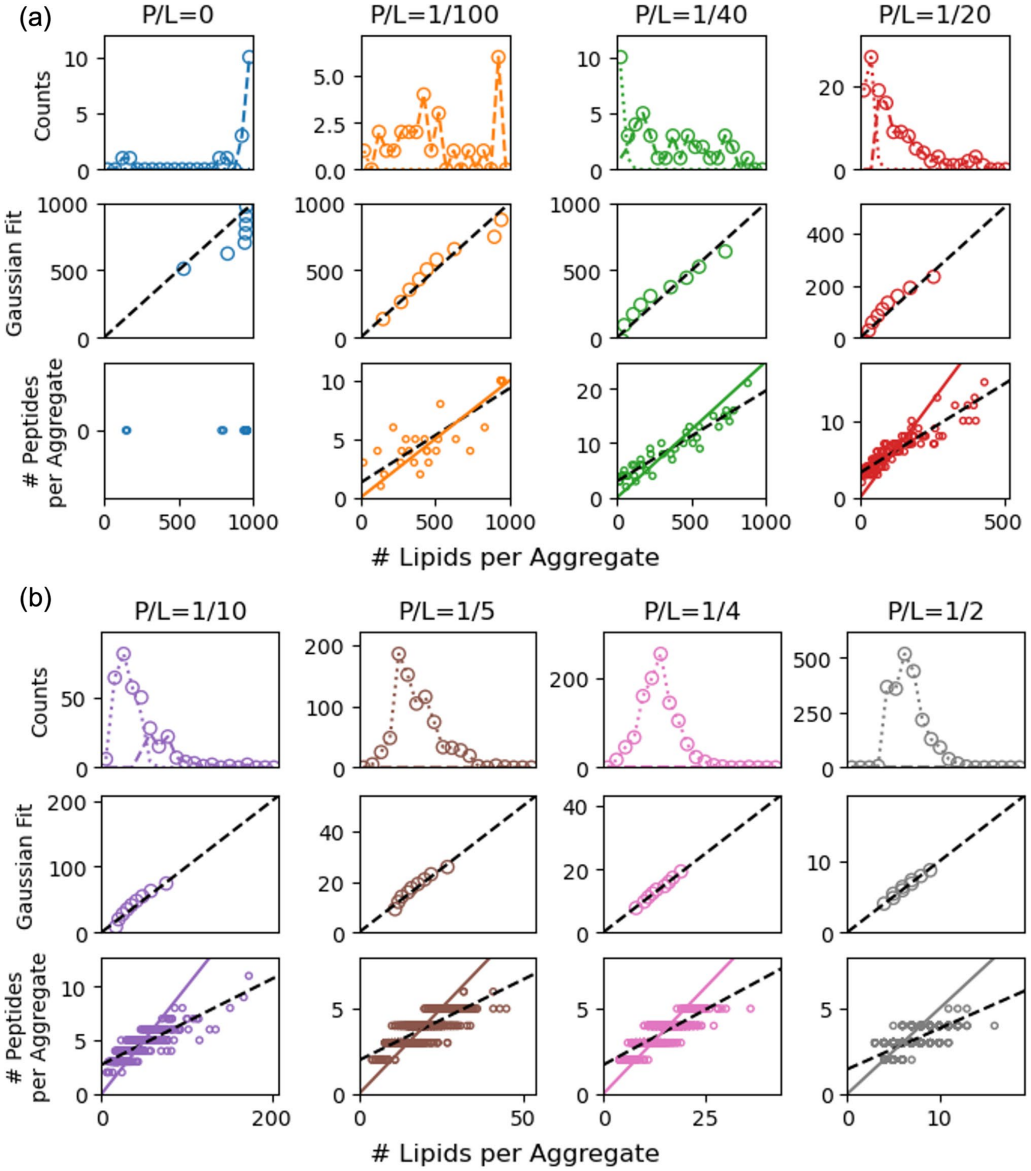
Histograms of number of lipids per aggregate, QQ-plots of Gaussian fit to lipid per aggregate distributions, scatter plots of the number of peptides and number of lipids per aggregate with the bulk P/L ratio (solid line) and fit (dashed) for eight P/L ratios. Four systems with definite or minimal finite-size effects (**a**) and no finite-size effects (**b**)

The distribution of aggregate size shown in the first rows of Fig. 5a and b depends on P/L. Generally, high P/L systems form smaller, normally distributed aggregates. Histograms of the lipids per aggregate were calculated and a corresponding comparison to Gaussian behavior was carried out using QQ-plots (middle rows of Fig. 5a and b) of the statistics calculated from the aggregates in final frame of all 15 replicas. The behavior at low lipid number is non-Gaussian because a Gaussian fit allows for negative lipids per aggregate. Separately, deviations from Gaussian behavior at high number of lipids are physically meaningful. Micelles produce Gaussian distributions of lipids per aggregate (e.g., P/L = 1/4 or 1/2) and deviations from Gaussian behavior are due to the emergence of discs. P/L = 1/40, 1/20, 1/10, and 1/5 systems form disc-like aggregates, and the distributions have corresponding long tails. In the lowest P/L system, two aggregates form on average, and the resulting distribution of lipids per aggregate peaks near the size of the system. The finite-size of the simulations results in a truncated distribution of lipids per aggregate and a poor Gaussian fit. Indeed, the deviations from Gaussian behavior are more pronounced in the pure lipid case, where there is about one aggregate in the system and is composed of most of the lipids in the system (see “Cooke Model: Lipids Only” subsection).

**Fig. 6:**
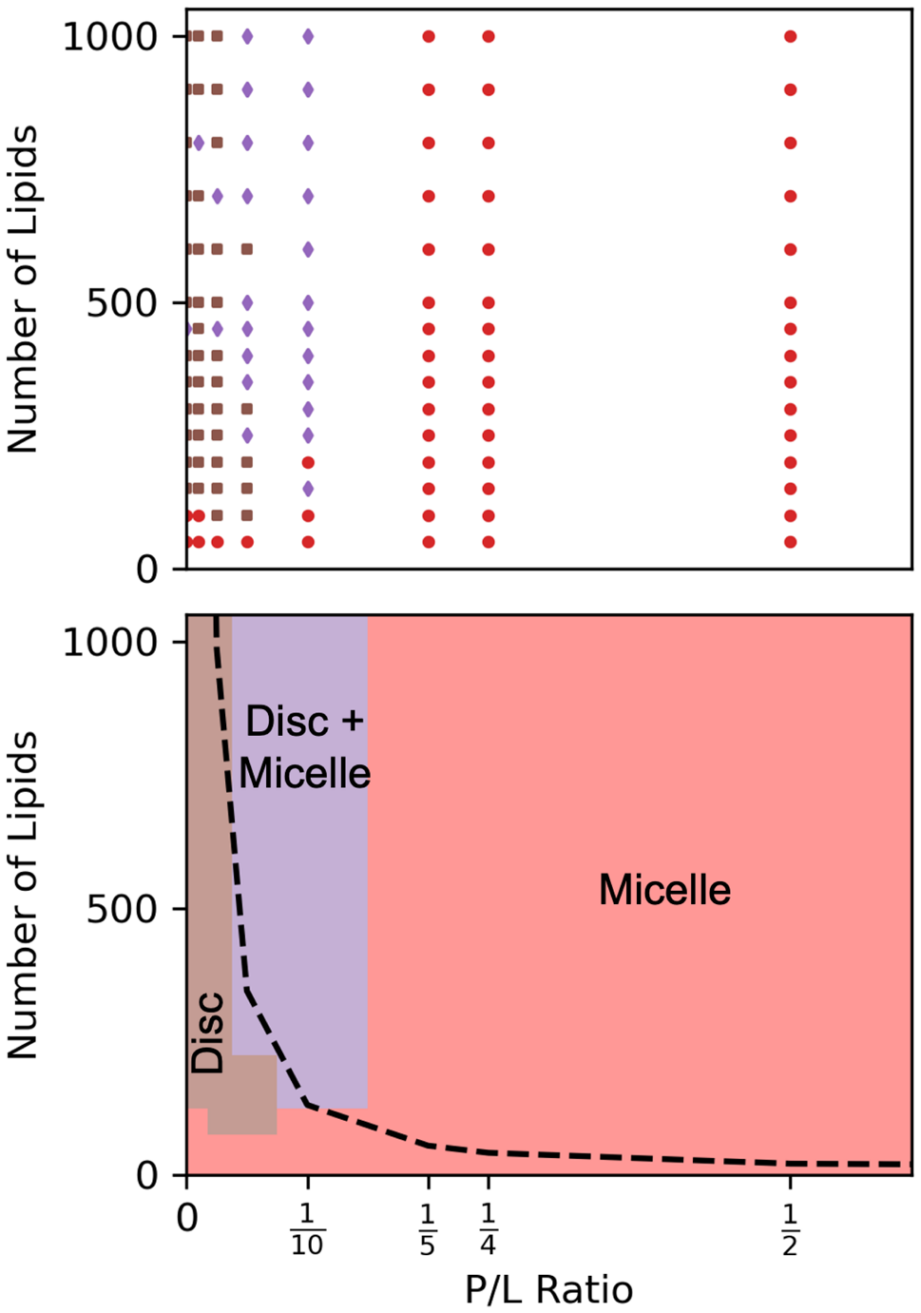
Finite-size driven phase diagram with the raw data (top) and smoothed data (bottom), where red circles are systems that only form micelles, brown rectangles form only discs, and purple diamonds are a mixture of discs and micelles. The black dashed line represents three times the preferred aggregate size and systems below the dashed line have finite-size effects

Figure 6 summarizes the finite-size driven phase behavior of this peptide/lipid model, based on single replica simulations every 50 lipids between 50 and 500 and every 100 lipids between 500 and 1000, and eight values of P/L (see Table S1 for full list). As already discussed, the systems with the lowest P/L exhibit the clearest finite-size effects. Obviously, the micelles formed with just 50 lipids in the system (shown as red circles in the bottom left of Fig. 6 top) form due to finite-size effects. Indeed, the systems with P/L = 0, 1/100, and 1/40 show finite-size effects in the relationship of number of aggregates and number of lipids per aggregate with system size. As shown in Figure S2, there is a plateau in the number of aggregates and a linear dependence of average number of lipids per aggregate with system size demonstrating finite-size effects in the lowest P/L ratios. With increasing system size, the intermediate P/L systems assemble into discs (brown rectangles), or a mixture of discs and micelles (purple diamonds) shown in Fig. 6a. The average number of lipids per aggregate in the systems at intermediate P/L oscillates with system size. P/L = 1/20 is the best example of these oscillations. Shown in Figure S2, the preferred number of lipids per aggregate is slightly above 100. As such, systems containing 100, 200, 300… lipids systematically underpredict the average number of lipids per aggregate and systems containing 150, 250, 350… lipids systematically overpredict the average number of lipids per aggregate. This behavior is more pronounced with sparse sampling of system size using a single replica as compared to Fig. 3, which reports a narrower range of system sizes.

P/L = 1/2 and 1/4 have already overcome any finite-size effects and consistently form micelles shown as red circles in Fig. 6 top. These provide an important contrast to those with possible finite-size effects. Figure 6 bottom presents a smoothed version of the preceding data, and delineates the region dominated by finite-size effect with a dashed line.

### Martini 3: DMPC and ELK-neu

**Fig. 7:**
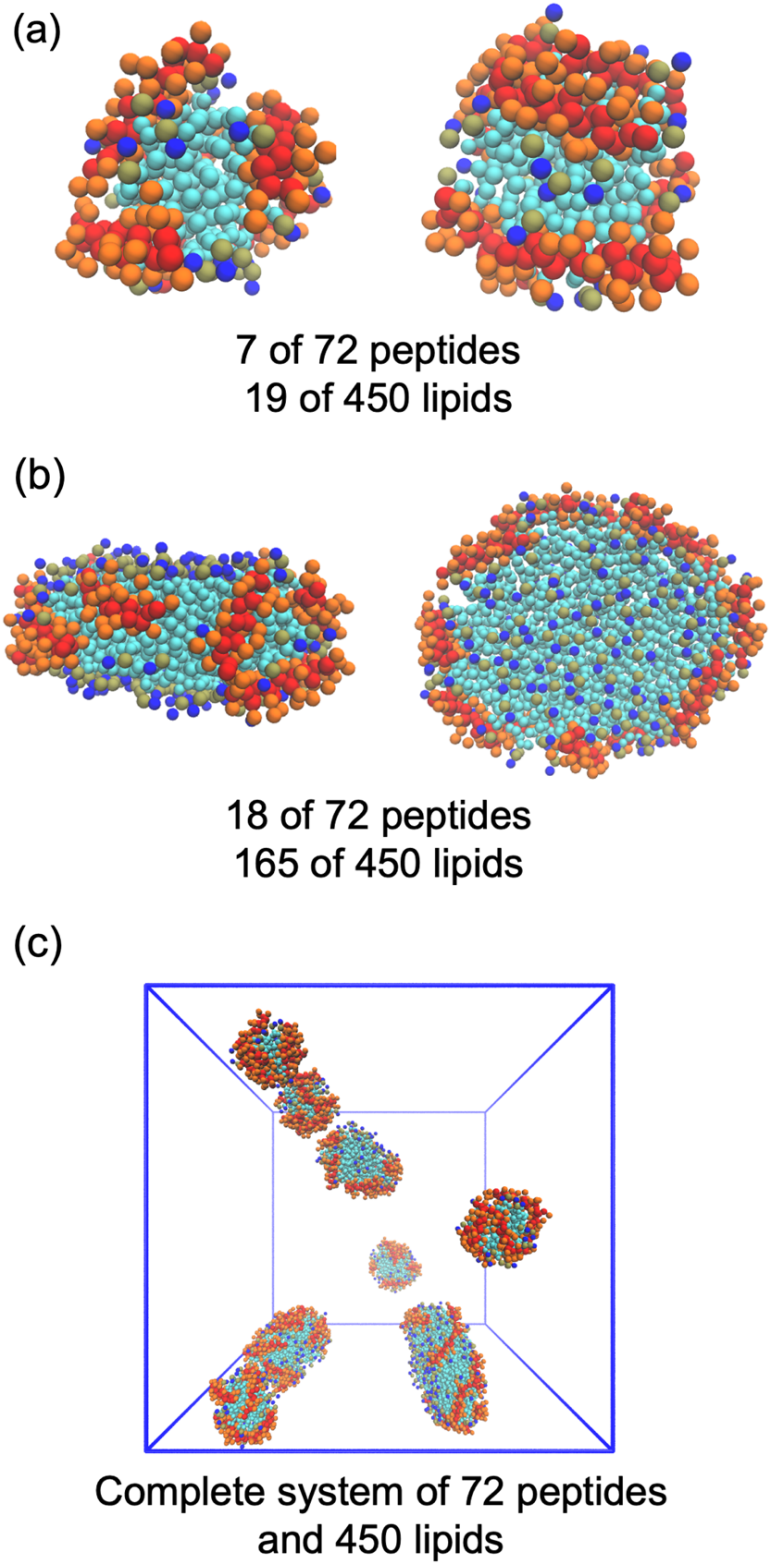
Representative snapshots of Martini 3 simulations. **a** two snapshots of the same mixed micelle composed of 7 peptides and 19 lipids and **b** top and side view disc composed of 18 peptides and 165 lipids in **c** 72-peptide and 450-lipid system excluding solvent. ELK-neu backbone and side chains shown in red and orange, hydrophobic beads of the lipids shown in cyan, phosphate shown in gold, and choline bead shown in blue

**Fig. 8:**
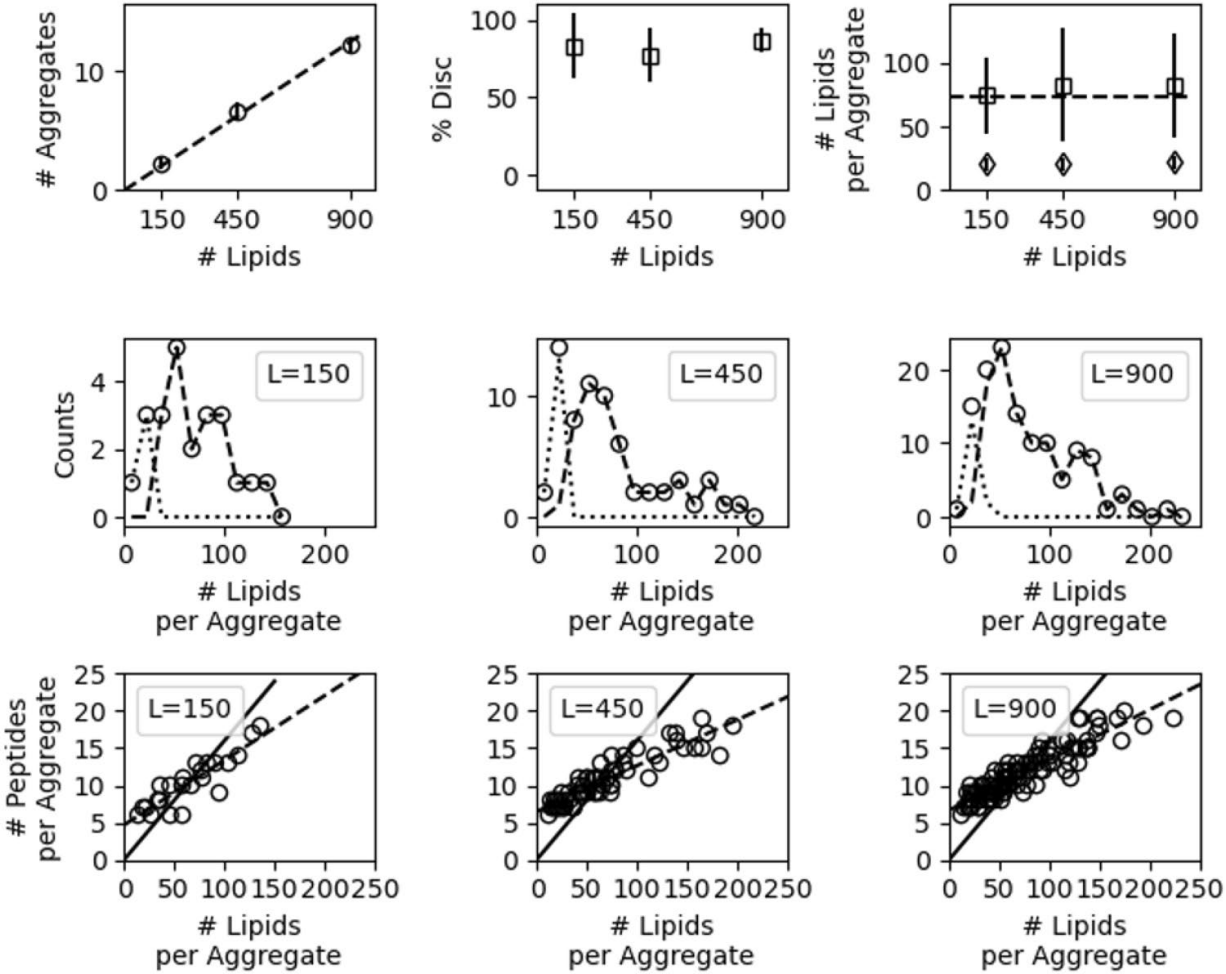
Top row: number of aggregates (left), percent of aggregates that are discs (center), and the number of lipids per disc and micelle (right) as a function of total number of lipids. Center row: histograms of the number of lipids per micelle (dotted) and disc (dashed) for the three system sizes. Bottom row: scatter plots of the number of peptides vs. number of lipids per aggregate with bulk P/L ratio as a solid line and a fit line as a dashed line for the three different system sizes

The final test of finite-size effects in two-component aggregation is the self-assembly of ELK-neu and DMPC for three system sizes: 150, 450, and 900 lipids, P/L = 1/6.25. This P/L is the same as experiments of ELK-neu in POPC and cholesterol and all-atom simulations of a single nanodisc composed of 24:150:15 ELK-neu:POPC:cholesterol. (Islam et al., 2018) Based on the preceding simulations, the system containing 24 ELK-neu and 150 DMPC was chosen as the smallest system size; i.e., if aggregation in DMPC were identical to that in POPC/cholesterol, there should be a single well-formed disc. However, in the ten replicas there were consistently two or three stable aggregates in a system of 150 lipids. The two or three aggregates frequently interacted with each other but did not coalesce suggesting these aggregates are stable over long-time simulations. Similarly, in the 450- and 900-lipid systems, the number of aggregates quickly decays to a similar number between the replicas as reflected by the ergodic measure (Fig. S3). The equilibrium aggregates are a mixture of micelles and discs. For example, in a single 450-lipid system shown in Fig. 7, a mixture of micelles and large discs form. The scatter plot of lipids and peptides per aggregate in the bottom row of Fig. 8 shows that the smaller, micellar aggregates have a higher P/L than bulk ratio (solid line) while the discs contain a lower P/L ratio than bulk and lie below the bulk ratio. The micelles are rich in peptide and have a higher P/L ratio than the bulk P/L ratio, and the discs are lipid-rich.

As shown in Fig. 8, finite-size effects are not appreciable for the three system sizes. The number of aggregates observed depends linearly on the system size, and the percent of discs is similar. However, the 150-lipid system exhibits slight finite-size effects because it cannot assemble into large aggregates seen in the 450- and 900-lipid systems, and therefore does not exhibit the same long tail in the distribution of lipids per aggregate. The truncated distribution resulted in slightly smaller average aggregate; the average aggregate diameter is 7.0, 7.1, and 7.4 nm for the 150-, 450-, and 900-lipid systems, respectively.

Taken together, the behavior of the ELK-neu/DMPC systems is like a Cooke system that forms a mixture of micelles and discs and has overcome considerable finite-size effects that affect the dominant phase. Specifically, the Cooke systems with P/L = 1/10 and 1/20 (Fig. 3) form a single micelle when the system is sufficiently small. With increasing system size, a mixture of micelles and discs forms and the average number of lipids per disc is no longer affected by system size. There is a plateau in the average lipids per disc with significant variance. Given this similarity in the Cooke and Martini systems, one can infer that the ELK-neu/DMPC simulations have overcome finite-size effects and produce a reliable estimate of disc and micelle size.

## Discussion

The goal of this study was to test common diagnoses for finite-size effects in the self-assembly of lipid/peptide systems and characterize the assembly of micelles and discs using the Cooke and Martini 3 models. The low-resolution and computationally inexpensive Cooke model was used to explore a wide range of system sizes and compositions. The pure lipid and low P/L systems form large discs with definite finite-size effects in all system sizes (i.e., those containing 1000 or fewer lipids), which contrasts with the high P/L systems that form micelles and overcome finite-size in relatively small systems. The Cooke model is a useful tool for testing finite-size effects, as summarized in a finite-size phase diagram (Fig. 6) that shows that P/L and system size directly affects the formation of discs, micelles, and mixtures. Next, insights from the Cooke model were applied to the higher resolution MartiniI 3 model. Simulations of three different system sizes showed that aggregate number scales with system size and that aggregate sizes are within statistical error of each other indicating minimal finite-size effects. However, the distribution of lipids per aggregate (i.e., aggregate size) from the 150-lipid shows a truncated tail that slightly reduces the average size of an aggregate. Thus, while self-assembly simulations of multicomponent discs are inherently different from homogenous micelle formation, finite-size effects are still largely overcome in systems that are three times the equilibrium aggregate size.

Previous studies on the finite-size effects in simulations of micelle self-assembly provide practical and theoretical context for the current study, but, to date, have not shown that the single-component heuristics are applicable to more complex systems. These include zwitterionic surfactants, ionic surfactants, and model data for a micelle-forming system, and demonstrate finite-size effects manifest in the dependence of the critical micelle concentration, the number of surfactants per aggregate and total number of aggregates on the total number of surfactants. (Harris et al., 2021; Kindt, 2013; Zhang et al., 2017) The resulting guidance is that finite-size effects are overcome when the system contains enough monomers to form at least three or five micelles of the preferred size. (Harris et al., 2021; Johnston et al., 2016) Results for the present simulations of lipid/peptide assembly, a two-component system with multiple phases, are consistent with and support further use of this heuristic.

In high P/L systems when only micelles form (i.e., no discs), simulations using the Cooke model do not have finite-size effects when the system size is three times larger than the equilibrium aggregate size. The behavior is like the previously studied two-component assembly of sodium and octyl sulfate and would be a possible application of Partition-Enabled Analysis of Cluster Histograms (PEACH) approach, which can extrapolate the 2D free energy surface ion/surfactant per aggregate from relatively small simulations of only one or two aggregates. (Zhang et al., 2017) On the other hand, the pure lipid or low P/L ratios are likely pathological cases for the PEACH approach as the preferred aggregate size is not reached within the system sizes modeled here and would be expected to fail to extrapolate to the infinite case. The systems with intermediate P/L ratios are unclear cases as these represent a two-phase system for which PEACH has not yet been applied.

Using the Cooke model to explore the finite-size effects and phase behavior of a broad range of P/L ratios is a new use of the model, but the observation of micelle/disc mixtures is not novel. Notably, Midtgaard et al. parameterized a Cooke model of ApoA1 mimetic peptides with significant polydispersity (i.e., range of self-assembled disc sizes) in agreement with small-angle neutron scattering and small-angle x-ray scattering experiments as well as found the formation of peptide trimers and peptide filaments. (Midtgaard et al., 2014) The peptide trimer aggregates formed by the ApoA1 mimetic are somewhat similar to the mixed micelles observed here, though the present peptide model forms a broader range of micelle sizes and P/L ratio per aggregate. Another key difference is the double-belt behavior of the ApoA1 mimetic, which is similar to the double-belt conformation of ApoA1 dimers that stabilize nascent high-density lipoprotein. (Pourmousa et al., 2018; Segrest et al., 1999) The current peptides, in contrast, arrange perpendicular to the flat surfaces, in what has been termed a “picket fence” and observed in simulations of ELK-neu in higher resolution models. (Islam et al., 2018) Furthermore, the double-belt arrangement of the ApoA1 mimetic required explicit tuning of peptide-peptide interactions. The current peptide model could similarly be refined via peptide-peptide interactions to optimize the peptide ordering. Additionally, the peptide shape and size (e.g., excluded volume) could be optimized using experimental, all-atom, and coarse-grained properties of ELK-neu as a potential target. For example, the current Cooke peptide is 14 times larger than a Cooke lipid while the Martini 3 representation of ELK-neu is about three times larger than DMPC. The overestimation of the peptide size could lead to an underestimation of the preferred peptides per aggregate. However, as the current model and that of Midtgaard et al. show, dedicated optimization of these peptide properties was not necessary to generate coexistence of micelles and discs.

The Martini 3 model of ELK-neu successfully solubilizes lipids and forms a wide range of peptide-lipid aggregate sizes. Gel electrophoresis measurements determined that the size of ELK-neu/DMPC aggregates is between 7 and 9 nm. (Islam et al., 2018) A naïve comparison to experiment would be to calculate the average size of all aggregates, which is 7.4 nm for the 900-lipid system. However, this estimate is biased by the inclusion of small micelles, which are not recorded in the experiment. The estimate of the average size of the self-assembled discs is 7.7 nm and is in better agreement with experiment. Recall that the P/L = 1/6.25 used here is based on measurements in POPC/cholesterol (see Methods), which may contribute to differences in simulation and experimental disc dimensions. In addition to a size estimate self-assembly simulations can provide estimates of the disc composition range; P/L for the 7 and 9 nm aggregates are 1/5.4 and 1/8.9, respectively.

In the case when the composition of the discs is known precisely (e.g., P/L = 1/6.25 in the ELK-neu/POPC/cholesterol cryo-EM experiment), self-assembly simulations can determine the size distribution of aggregates with that precise composition. For the 900-lipid system, the size of the self-assembled discs with P/L = 1/6.25 is 7.7 nm, which excludes both the small, high P/L micelles and the large, low P/L discs generated in the simulation. Indeed, the distribution of disc size resembles the distribution from experimental measurement of the ELK-neu/POPC/cholesterol, which also has a long tail. Thus, the Martini 3 model is an overall useful tool for providing approximate disc size and composition relative to experimental results.

## Conclusions

Finite-size effects in self-assembly simulations of lipids and peptides can affect aggregate phase, size, and critical micelle concentration. Heuristics based on single-component assembly suggest that finite-size effects should be overcome in systems that are at least three to five times larger than the preferred aggregate size. Indeed, CG simulations using the Cooke lipid model and a new compatible ELK-neu model show that finite-size effects on phase and aggregate size are overcome in systems three times the equilibrium aggregate size. The estimate of the average aggregate size is complicated by the formation of lipid/peptide discs, which produce a long tail in the distribution of lipids per aggregate. At intermediate P/L ratios, a mixture of micelles and discs forms with the average disc containing many more lipids than the average micelle. Thus, finite-size effects can result in an underestimate of the preferred aggregate size. In small systems, the preferred aggregate is a micelle, but in fact, large discs form when the system is sufficiently large and skew estimates of the equilibrium aggregate size. Simulations of DMPC/ELK-neu using the Martini 3 model showed similar behavior. The smallest sized simulation containing only 150 lipids could not form the largest aggregates seen in simulations containing 450 and 900 lipids. However, the three systems produced similar estimates of the size of an aggregate, all within the range of aggregate sizes measured experimentally. Overall, the Martini 3 model proved to be a viable model to estimate the size of ELK-neu/DMPC aggregates given sufficient system size.

## Supplementary Information

Below is the link to the electronic supplementary material.

Supplementary file1 (DOCX 612 kb)

## Data Availability

The datasets generated during and/or analyzed during the current study are available from the corresponding author on reasonable request.
