# Supplementary material for: Finite-Size Effects in Simulations of Peptide/Lipid Assembly
Source: J Membr Biol. 2022 Jul 19;255(4-5):437–49. doi: 10.1007/s00232-022-00255-9 (PMC9581812; doi:10.1007/s00232-022-00255-9)
Supplement: Supplementary file 1 — Supplementary file1 (DOCX 612 kb) [file 232_2022_255_MOESM1_ESM.docx]

**Supplemental Information for “Finite-size effects in simulations of peptide/lipid assembly”**

Zack Jarin, Olivia Agolini, and Richard W. Pastor*

Laboratory of Computational Biology, National Heart, Lung, and Blood Institute, National Institutes of Health, Bethesda, Maryland

* Corresponding Author: pastorr@nhlbi.nih.gov, orcid:0000-0002-2454-5131

Figure S1: Number of aggregates and ergodic measure a function of time for 1,000-lipid systems and eight P/L ratios. The number of aggregates is averaged over 15 replicas and the standard deviation is shown in the shaded region. The ergodic measure is an instantaneous value calculated from the number of aggregates in the same 15 replicas.


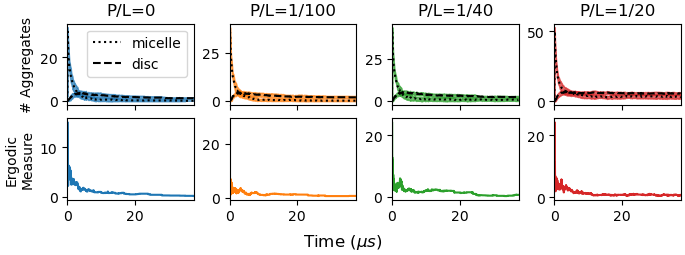

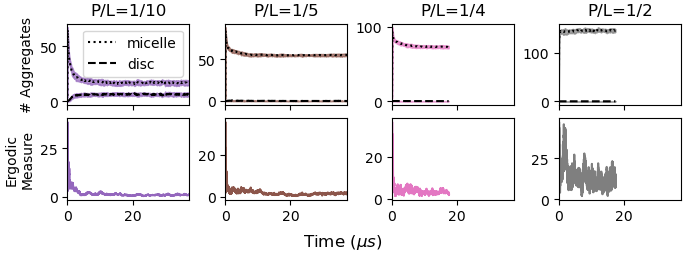


(a)

(b)

Figure S2: Number of aggregates and number of lipids per aggregates as a function of number of lipids for eight P/L ratios. The slope of fit to the number of aggregates vs. number of lipids is the average number of lipids per aggregate.


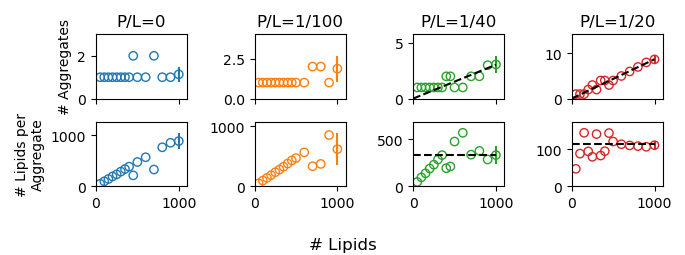

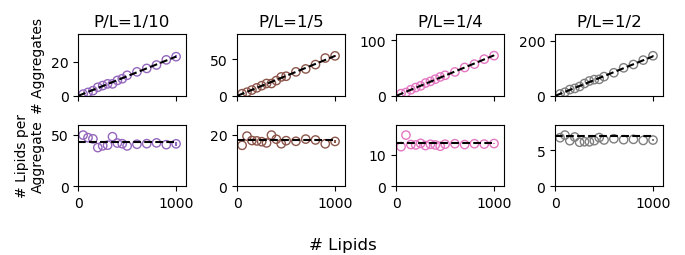


(a)

(b)


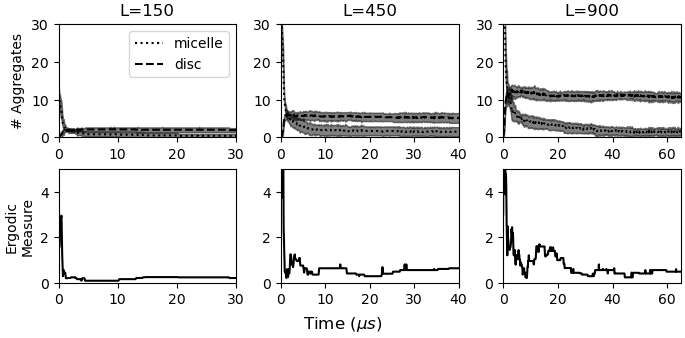
Figure S3: Timeseries of the number of aggregates and the ergodic measure for the three MARTINI 3 systems containing 150, 450, and 900 lipids.

SI Table 1: System details of simulations included in Figures 2a, 4, 5, and 6 as well as S1 and S2. Single replicas of all simulations were run, except the 15 replicas of the 1,000-lipid systems.

| Number of Lipids | Number of Peptides | P/L Ratio | Box Volume (nm^3^) | Run Time (μs) |
| --- | --- | --- | --- | --- |
| 50 | 0 | 0 | 8.30E+03 | 17.5 |
| 50 | 1 | 1/40 | 8.30E+03 | 17.5 |
| 50 | 2 | 1/20 | 8.30E+03 | 17.5 |
| 50 | 5 | 1/10 | 8.30E+03 | 17.5 |
| 50 | 10 | 1/5 | 8.30E+03 | 17.5 |
| 50 | 12 | 1/4 | 8.30E+03 | 17.5 |
| 50 | 25 | 1/2 | 8.30E+03 | 17.5 |
|  |  |  |  |  |
| 100 | 0 | 0 | 1.66E+04 | 17.5 |
| 100 | 1 | 1/100 | 1.66E+04 | 17.5 |
| 100 | 2 | 1/40 | 1.66E+04 | 17.5 |
| 100 | 5 | 1/20 | 1.66E+04 | 17.5 |
| 100 | 10 | 1/10 | 1.66E+04 | 17.5 |
| 100 | 20 | 1/5 | 1.66E+04 | 17.5 |
| 100 | 25 | 1/4 | 1.66E+04 | 17.5 |
| 100 | 50 | 1/2 | 1.66E+04 | 17.5 |
|  |  |  |  |  |
| 150 | 0 | 0 | 2.49E+04 | 17.5 |
| 150 | 1 | 1/100 | 2.49E+04 | 17.5 |
| 150 | 3 | 1/40 | 2.49E+04 | 17.5 |
| 150 | 7 | 1/20 | 2.49E+04 | 17.5 |
| 150 | 15 | 1/10 | 2.49E+04 | 17.5 |
| 150 | 30 | 1/5 | 2.49E+04 | 17.5 |
| 150 | 37 | 1/4 | 2.49E+04 | 17.5 |
| 150 | 75 | 1/2 | 2.49E+04 | 17.5 |
|  |  |  |  |  |
| 200 | 0 | 0 | 3.32E+04 | 17.5 |
| 200 | 2 | 1/100 | 3.32E+04 | 17.5 |
| 200 | 5 | 1/40 | 3.32E+04 | 17.5 |
| 200 | 10 | 1/20 | 3.32E+04 | 17.5 |
| 200 | 20 | 1/10 | 3.32E+04 | 17.5 |
| 200 | 40 | 1/5 | 3.32E+04 | 17.5 |
| 200 | 50 | 1/4 | 3.32E+04 | 17.5 |
| 200 | 100 | 1/2 | 3.32E+04 | 17.5 |
|  |  |  |  |  |
| 250 | 0 | 0 | 4.15E+04 | 17.5 |
| 250 | 2 | 1/100 | 4.15E+04 | 17.5 |
| 250 | 6 | 1/40 | 4.15E+04 | 17.5 |
| 250 | 12 | 1/20 | 4.15E+04 | 17.5 |
| 250 | 25 | 1/10 | 4.15E+04 | 17.5 |
| 250 | 50 | 1/5 | 4.15E+04 | 17.5 |
| 250 | 62 | 1/4 | 4.15E+04 | 17.5 |
| 250 | 125 | 1/2 | 4.15E+04 | 17.5 |
|  |  |  |  |  |
| 300 | 0 | 0 | 4.98E+04 | 17.5 |
| 300 | 3 | 1/100 | 4.98E+04 | 17.5 |
| 300 | 7 | 1/40 | 4.98E+04 | 17.5 |
| 300 | 15 | 1/20 | 4.98E+04 | 17.5 |
| 300 | 30 | 1/10 | 4.98E+04 | 17.5 |
| 300 | 60 | 1/5 | 4.98E+04 | 17.5 |
| 300 | 75 | 1/4 | 4.98E+04 | 17.5 |
| 300 | 150 | 1/2 | 4.98E+04 | 17.5 |
|  |  |  |  |  |
| 350 | 0 | 0 | 5.81E+04 | 17.5 |
| 350 | 3 | 1/100 | 5.81E+04 | 17.5 |
| 350 | 8 | 1/40 | 5.81E+04 | 17.5 |
| 350 | 17 | 1/20 | 5.81E+04 | 17.5 |
| 350 | 35 | 1/10 | 5.81E+04 | 17.5 |
| 350 | 70 | 1/5 | 5.81E+04 | 17.5 |
| 350 | 87 | 1/4 | 5.81E+04 | 17.5 |
| 350 | 175 | 1/2 | 5.81E+04 | 17.5 |
|  |  |  |  |  |
| 400 | 0 | 0 | 6.64E+04 | 17.5 |
| 400 | 4 | 1/100 | 6.64E+04 | 17.5 |
| 400 | 10 | 1/40 | 6.64E+04 | 17.5 |
| 400 | 20 | 1/20 | 6.64E+04 | 17.5 |
| 400 | 40 | 1/10 | 6.64E+04 | 17.5 |
| 400 | 80 | 1/5 | 6.64E+04 | 17.5 |
| 400 | 100 | 1/4 | 6.64E+04 | 17.5 |
| 400 | 200 | 1/2 | 6.64E+04 | 17.5 |
|  |  |  |  |  |
| 450 | 0 | 0 | 7.47E+04 | 17.5 |
| 450 | 4 | 1/100 | 7.47E+04 | 17.5 |
| 450 | 11 | 1/40 | 7.47E+04 | 17.5 |
| 450 | 22 | 1/20 | 7.47E+04 | 17.5 |
| 450 | 45 | 1/10 | 7.47E+04 | 17.5 |
| 450 | 90 | 1/5 | 7.47E+04 | 17.5 |
| 450 | 112 | 1/4 | 7.47E+04 | 17.5 |
| 450 | 225 | 1/2 | 7.47E+04 | 17.5 |
|  |  |  |  |  |
| 500 | 0 | 0 | 8.30E+04 | 37.5 |
| 500 | 5 | 1/100 | 8.30E+04 | 37.5 |
| 500 | 12 | 1/40 | 8.30E+04 | 37.5 |
| 500 | 25 | 1/20 | 8.30E+04 | 37.5 |
| 500 | 50 | 1/10 | 8.30E+04 | 37.5 |
| 500 | 100 | 1/5 | 8.30E+04 | 37.5 |
| 500 | 125 | 1/4 | 8.30E+04 | 37.5 |
| 500 | 250 | 1/2 | 8.30E+04 | 37.5 |
|  |  |  |  |  |
| 600 | 0 | 0 | 9.96E+04 | 37.5 |
| 600 | 6 | 1/100 | 9.96E+04 | 37.5 |
| 600 | 15 | 1/40 | 9.96E+04 | 37.5 |
| 600 | 30 | 1/20 | 9.96E+04 | 37.5 |
| 600 | 60 | 1/10 | 9.96E+04 | 37.5 |
| 600 | 120 | 1/5 | 9.96E+04 | 37.5 |
| 600 | 150 | 1/4 | 9.96E+04 | 37.5 |
| 600 | 300 | 1/2 | 9.96E+04 | 37.5 |
|  |  |  |  |  |
| 700 | 0 | 0 | 1.16E+05 | 37.5 |
| 700 | 7 | 1/100 | 1.16E+05 | 37.5 |
| 700 | 17 | 1/40 | 1.16E+05 | 37.5 |
| 700 | 35 | 1/20 | 1.16E+05 | 37.5 |
| 700 | 70 | 1/10 | 1.16E+05 | 37.5 |
| 700 | 140 | 1/5 | 1.16E+05 | 37.5 |
| 700 | 175 | 1/4 | 1.16E+05 | 37.5 |
| 700 | 350 | 1/2 | 1.16E+05 | 37.5 |
|  |  |  |  |  |
| 800 | 0 | 0 | 1.33E+05 | 37.5 |
| 800 | 8 | 1/100 | 1.33E+05 | 37.5 |
| 800 | 20 | 1/40 | 1.33E+05 | 37.5 |
| 800 | 40 | 1/20 | 1.33E+05 | 37.5 |
| 800 | 80 | 1/10 | 1.33E+05 | 37.5 |
| 800 | 160 | 1/5 | 1.33E+05 | 37.5 |
| 800 | 200 | 1/4 | 1.33E+05 | 37.5 |
| 800 | 400 | 1/2 | 1.33E+05 | 37.5 |
|  |  |  |  |  |
| 900 | 0 | 0 | 1.49E+05 | 37.5 |
| 900 | 9 | 1/100 | 1.49E+05 | 37.5 |
| 900 | 22 | 1/40 | 1.49E+05 | 37.5 |
| 900 | 45 | 1/20 | 1.49E+05 | 37.5 |
| 900 | 90 | 1/10 | 1.49E+05 | 37.5 |
| 900 | 180 | 1/5 | 1.49E+05 | 37.5 |
| 900 | 225 | 1/4 | 1.49E+05 | 37.5 |
| 900 | 450 | 1/2 | 1.49E+05 | 37.5 |
|  |  |  |  |  |
| 1000 | 0 | 0 | 1.66E+05 | 37.5 × 15 |
| 1000 | 10 | 1/100 | 1.66E+05 | 37.5 × 15 |
| 1000 | 25 | 1/40 | 1.66E+05 | 37.5 × 15 |
| 1000 | 50 | 1/20 | 1.66E+05 | 37.5 × 15 |
| 1000 | 100 | 1/10 | 1.66E+05 | 37.5 × 15 |
| 1000 | 200 | 1/5 | 1.66E+05 | 37.5 × 15 |
| 1000 | 250 | 1/4 | 1.66E+05 | 37.5 × 15 |
| 1000 | 500 | 1/2 | 1.66E+05 | 37.5 × 15 |

Table S2: Simulation details of system in Figure 2b. Single replicas of all systems were run except 15 replicas of the 10^-2^ M system were run and are the same 1,000-lipid, P/L=0 simulations in Table S1.

| Number of Lipids | Concentration (mol/L) | Box Volume (nm^3^) | Run Time (μs) |
| --- | --- | --- | --- |
| 1000 | 1.00E-01 | 1.66E+04 | 37.5 |
| 1000 | 1.00E-02 | 1.66E+05 | 37.5 × 15 |
| 1000 | 9.00E-03 | 1.85E+05 | 37.5 |
| 1000 | 8.00E-03 | 2.98E+05 | 37.5 |
| 1000 | 7.00E-03 | 2.37E+05 | 37.5 |
| 1000 | 6.00E-03 | 2.77E+05 | 37.5 |
| 1000 | 5.00E-03 | 3.32E+05 | 37.5 |
| 1000 | 4.00E-03 | 4.15E+06 | 37.5 |
| 1000 | 3.00E-03 | 5.54E+06 | 37.5 |
| 1000 | 2.00E-03 | 8.30E+05 | 37.5 |
| 1000 | 1.00E-03 | 1.66E+06 | 37.5 |
| 1000 | 9.00E-04 | 1.85E+06 | 37.5 |
| 1000 | 8.00E-04 | 2.08E+06 | 37.5 |
| 1000 | 7.00E-04 | 2.37E+06 | 37.5 |
| 1000 | 6.00E-04 | 2.77E+06 | 37.5 |
| 1000 | 5.00E-04 | 3.32E+06 | 37.5 |
| 1000 | 4.00E-04 | 4.15E+06 | 37.5 |
| 1000 | 3.00E-04 | 5.54E+06 | 37.5 |
| 1000 | 2.00E-04 | 8.30E+06 | 37.5 |
| 1000 | 1.00E-04 | 1.66E+07 | 37.5 |
| 1000 | 1.00E-05 | 1.66E+08 | 37.5 |

SI Table 3: Simulation details of systems in Figure 3. 10 replicas of each system were run.

| Number of Lipids | Number of Peptides | P/L Ratio | Box Volume (nm^3^) | Run Time μs) |
| --- | --- | --- | --- | --- |
| 40 | 2 | 1/20 | 6.64E+03 | 17.5 × 10 |
| 80 | 4 | 1/20 | 1.33E+04 | 17.5 × 10 |
| 120 | 6 | 1/20 | 1.99E+04 | 17.5 × 10 |
| 160 | 8 | 1/20 | 2.66E+04 | 17.5 × 10 |
| 200 | 10 | 1/20 | 3.32E+04 | 17.5 × 10 |
| 240 | 12 | 1/20 | 3.99E+04 | 17.5 × 10 |
| 280 | 14 | 1/20 | 4.65E+04 | 17.5 × 10 |
| 320 | 16 | 1/20 | 5.31E+04 | 17.5 × 10 |
| 360 | 18 | 1/20 | 5.98E+04 | 17.5 × 10 |
| 400 | 20 | 1/20 | 6.64E+04 | 17.5 × 10 |
| 440 | 22 | 1/20 | 7.31E+04 | 17.5 × 10 |
| 480 | 24 | 1/20 | 7.97E+04 | 17.5 × 10 |
| 520 | 26 | 1/20 | 8.64E+04 | 37.5 × 10 |
| 560 | 28 | 1/20 | 9.30E+04 | 37.5 × 10 |
| 600 | 30 | 1/20 | 9.96E+04 | 37.5 × 10 |
| 640 | 32 | 1/20 | 1.06E+05 | 37.5 × 10 |
| 680 | 34 | 1/20 | 1.13E+05 | 37.5 × 10 |
| 720 | 36 | 1/20 | 1.20E+05 | 37.5 × 10 |
| 760 | 38 | 1/20 | 1.26E+05 | 37.5 × 10 |
| 800 | 40 | 1/20 | 1.33E+05 | 37.5 × 10 |
|  |  |  |  |  |
| 20 | 2 | 1/10 | 3.32E+03 | 17.5 × 10 |
| 40 | 4 | 1/10 | 6.64E+03 | 17.5 × 10 |
| 60 | 6 | 1/10 | 9.96E+03 | 17.5 × 10 |
| 80 | 8 | 1/10 | 1.33E+04 | 17.5 × 10 |
| 100 | 10 | 1/10 | 1.66E+04 | 17.5 × 10 |
| 120 | 12 | 1/10 | 1.99E+04 | 17.5 × 10 |
| 140 | 14 | 1/10 | 2.32E+04 | 17.5 × 10 |
| 160 | 16 | 1/10 | 2.66E+04 | 17.5 × 10 |
| 180 | 18 | 1/10 | 2.99E+04 | 17.5 × 10 |
| 200 | 20 | 1/10 | 3.32E+04 | 17.5 × 10 |
| 220 | 22 | 1/10 | 3.65E+04 | 17.5 × 10 |
| 240 | 24 | 1/10 | 3.99E+04 | 17.5 × 10 |
| 260 | 26 | 1/10 | 4.32E+04 | 17.5 × 10 |
| 280 | 28 | 1/10 | 4.65E+04 | 17.5 × 10 |
| 300 | 30 | 1/10 | 4.98E+04 | 17.5 × 10 |
| 320 | 32 | 1/10 | 5.31E+04 | 17.5 × 10 |
| 340 | 34 | 1/10 | 5.65E+04 | 17.5 × 10 |
| 360 | 36 | 1/10 | 5.98E+04 | 17.5 × 10 |
| 380 | 38 | 1/10 | 6.31E+04 | 17.5 × 10 |
| 400 | 40 | 1/10 | 6.64E+04 | 17.5 × 10 |
|  |  |  |  |  |
| 10 | 2 | 1/5 | 1.66E+03 | 17.5 × 10 |
| 20 | 4 | 1/5 | 3.32E+03 | 17.5 × 10 |
| 30 | 6 | 1/5 | 4.98E+03 | 17.5 × 10 |
| 40 | 8 | 1/5 | 6.64E+03 | 17.5 × 10 |
| 50 | 10 | 1/5 | 8.30E+03 | 17.5 × 10 |
| 60 | 12 | 1/5 | 9.96E+03 | 17.5 × 10 |
| 70 | 14 | 1/5 | 1.16E+04 | 17.5 × 10 |
| 80 | 16 | 1/5 | 1.33E+04 | 17.5 × 10 |
| 90 | 18 | 1/5 | 1.49E+04 | 17.5 × 10 |
| 100 | 20 | 1/5 | 1.66E+04 | 17.5 × 10 |
| 110 | 22 | 1/5 | 1.83E+04 | 17.5 × 10 |
| 120 | 24 | 1/5 | 1.99E+04 | 17.5 × 10 |
| 130 | 26 | 1/5 | 2.16E+04 | 17.5 × 10 |
| 140 | 28 | 1/5 | 2.32E+04 | 17.5 × 10 |
| 150 | 30 | 1/5 | 2.49E+04 | 17.5 × 10 |
| 160 | 32 | 1/5 | 2.66E+04 | 17.5 × 10 |
| 170 | 34 | 1/5 | 2.82E+04 | 17.5 × 10 |
| 180 | 36 | 1/5 | 2.99E+04 | 17.5 × 10 |
| 190 | 38 | 1/5 | 3.16E+04 | 17.5 × 10 |
| 200 | 40 | 1/5 | 3.32E+04 | 17.5 × 10 |
|  |  |  |  |  |
| 4 | 2 | 1/2 | 6.64E+02 | 17.5 × 10 |
| 8 | 4 | 1/2 | 1.33E+03 | 17.5 × 10 |
| 12 | 6 | 1/2 | 1.99E+03 | 17.5 × 10 |
| 16 | 8 | 1/2 | 2.66E+03 | 17.5 × 10 |
| 20 | 10 | 1/2 | 3.32E+03 | 17.5 × 10 |
| 24 | 12 | 1/2 | 3.99E+03 | 17.5 × 10 |
| 28 | 14 | 1/2 | 4.65E+03 | 17.5 × 10 |
| 32 | 16 | 1/2 | 5.31E+03 | 17.5 × 10 |
| 36 | 18 | 1/2 | 5.98E+03 | 17.5 × 10 |
| 40 | 20 | 1/2 | 6.64E+03 | 17.5 × 10 |
|  |  |  |  |  |
|  |  |  |  |  |
|  |  |  |  |  |
